# Donor-derived cell-free DNA, gene expression profile, and acute rejection in donation after circulatory death (DCD) heart transplant recipients

**DOI:** 10.1016/j.jhlto.2024.100099

**Published:** 2024-04-23

**Authors:** Quan M. Bui, Yan Gernhofer, Antoinette S. Birs, Elizabeth Silver, Alessia Argiro, Benjamin Cruz, Eric Adler, Mark Kearns, Marcus A. Urey, Victor Pretorius

**Affiliations:** a Division of Cardiovascular Medicine, Department of Medicine, University of California, San Diego, La Jolla, California; b University of the Incarnate Word School of Osteopathic Medicine, San Antonio, Texas; c Cardiomyopathy Unit, University of Florence, Florence, Italy; d Division of Cardiovascular and Thoracic Surgery, Department of Surgery, University of California, San Diego, La Jolla, California

**Keywords:** heart transplant, donor-derived cell-free DNA, gene expression profile, donation after circulatory death, acute rejection

## Abstract

**Background:**

Acute rejection (AR) is a leading cause of early morbidity and mortality in heart transplant (HTx) recipients. There is limited data on donation after circulatory death (DCD) HTx recipients where ischemia reperfusion injury may contribute to the development of AR and may be detected early with noninvasive biomarkers, such as donor-derived cell-free DNA (dd-cfDNA) and gene expression profile (GEP). The goal of this study is to compare dd-cfDNA, GEP, and rejection outcomes in DCD and donation after brain death (DBD) HTx recipients.

**Methods:**

This single-center, retrospective study included DCD and DBD HTx recipients from January 2020 to September 2022. Patients were excluded from the study if dd-cfDNA and GEP were not available. The CareDx HeartCare platform was used to obtain dd-cfDNA (AlloSure-Heart) and GEP (AlloMap) with the highest values for each patient recorded at 6 and 12 months. The mean values for these noninvasive markers were compared between DCD and DBD groups. Patients were followed for clinical outcomes, including treated AR, cardiac allograft vasculopathy (CAV), and death, through September 2023.

**Results:**

A total of 156 HTx patients were included with 50 DCD and 106 DBD recipients. Baseline characteristics were similar between DCD and DBD recipients including mean age (58.5 vs 56.9 years, *p* = 0.48), male sex (82% vs 76%, *p* = 0.56), and Caucasian race (46% vs 43%, *p* = 0.78). There were no significant differences in mean AlloSure-Heart at 6 and 12 months between DCD and DBD recipients. AlloMap was significantly lower at 6 months (*p* = 0.04), but not at 12 months between DCD and DBD recipients. With respect to clinical outcomes at 1 year, there were no significant differences in treated AR (22% vs 14%, *p* = 0.32), International society of heart and lung transplant grade ≥2 CAV (0% vs 4%, *p* = 0.44), or mortality (2% vs 0%, *p* = 0.69) between DCD and DBD recipients.

**Conclusions:**

In this single-center study, there were no significant differences in mean dd-cfDNA and treated AR at 1 year between DCD and DBD HTx recipients. There was a significant increase in mean GEP at 6 months, but not at 12 months in the DBD cohort. Overall, there does not appear to be clinically significant cardiac allograft injury related to DCD as measured by noninvasive markers. Further work is needed to confirm these findings.

## Background

Heart transplant (HTx) remains the standard treatment for patients with advanced heart failure but is a limited resource. The use of donation after circulatory death (DCD) donors has increased the donor pool with early reports demonstrating comparable survival to donation after brain death (DBD) donors.^1^, ^2^ However, the obligatory ischemic time required with DCD may lead to significant morbidity with early reports demonstrating a higher incidence of moderate or severe primary graft dysfunction (PGD).^1^, ^3^ Furthermore, a recent study using the United Network for Organ Sharing database found that DCD HTx recipients were more likely to have acute rejection (AR) compared to DBD HTx recipients.^4^ Potential explanations for higher AR in DCD recipients include prolonged ischemic time, difference in procurement techniques, and higher rates of initial PGD that can all lead to upregulation of inflammatory markers and cardiac allograft injury.^4^

Noninvasive assays, including donor-derived cell-free DNA (dd-cfDNA) and gene expression profile (GEP), have become validated biomarkers to detect cardiac allograft injury and AR in HTx.^5^ Dd-cfDNA are short DNA fragments that are released into the recipient circulation with allograft injury and can be detected using sequencing and single nucleotide polymorphism assessment.^6^ GEP works by assessing immune activation in peripheral blood specimens using expression of a panel of genes. These noninvasive markers may be able to identify early cardiac allograft injury due to ischemia reperfusion injury and explain increased AR seen in DCD HTx recipients.

Currently, there is no data on dd-cfDNA and GEP in DCD HTx recipients. The goal of this study is to compare dd-cfDNA, GEP, and AR outcomes in DCD and DBD HTx recipients. Describing the trajectory and determining a baseline for these noninvasive markers in DCD HTx recipients may help provide insight into post-transplant morbidity, including AR and allograft injury.

## Materials and methods

This single-center, retrospective study included all patients who underwent HTx at the University of California, San Diego from January 2020 to September 2022. Patients were excluded from the study if dd-cfDNA and GEP were not available. Patients were also excluded if they were multiorgan transplant recipients and had either active pregnancy or malignancy as these conditions have been known to affect dd-cfDNA levels. The CareDx HeartCare platform was used to obtain dd-cfDNA (AlloSure) and GEP (AlloMap). The highest values were recorded at 6 and 12 months for each patient. The mean values for AlloSure and AlloMap at 6 and 12 months after transplant were compared as an aggregate between DCD and DCD HTx groups. Of note, the University of California, San Diego adult DCD HTx program started on September 12, 2020. The study was approved by the University of California, San Diego Human Research Protection Program.

### DCD heart transplant protocol

Two organ procurement techniques were used (1) direct procurement protocol with normothermic machine perfusion (DPP) and (2) thoraco-abdominal normothermic regional perfusion (TA-NRP). After withdrawal of life-sustaining therapy, donor organs were accepted up to 120 minutes for TA-NRP and up to 30 minutes for DPP as determined on a case-by-case basis. No changes to protocols were made throughout this study period.

### Immunosuppression protocol

With regards to perioperative immunosuppression, induction therapy is considered on a case-by-case basis. Indications for induction include but are not limited to sensitized patients (panel reactive antibody > 40%), renal insufficiency (GFR < 40), and African American (age < 40 years old). With regards to maintenance immunosuppression, the most common regimen consists of a calcineurin inhibitor, an antiproliferative agent, and a corticosteroid. For example, a typical regimen includes tacrolimus, mycophenolate mofetil, and prednisone. The corticosteroids are tapered slowly and usually discontinued after 6 months. Of note, our program introduces mammalian target of rapamycin inhibitors (i.e., sirolimus) early, usually within 1 year, to reduce risk of cardiac allograft vasculopathy (CAV) and calcineurin inhibitor-related toxicities.

### Rejection surveillance protocol

Our HTx rejection surveillance protocol consists of endomyocardial biopsy (EMBx) and HeartCare. Every patient undergoes EMBx biweekly for the first 3 months after transplant. Afterward, EMBx occurs monthly depending on AR risk: low (once at month 4), moderate (months 4-6), and high (months 4-10). Patients are risk stratified by the HTx team depending on the number of treated rejection episodes. The HeartCare protocol begins 1 month after transplant and is checked monthly until 1 year. After 1 year, HeartCare is checked less frequently but does not apply to this study.

### Outcomes

Patients were followed for clinical outcomes, including AR, CAV, and death, through September 2023. The primary outcome was biopsy-confirmed, AR requiring treatment within 1 year. Heart allograft pathology specimens are graded according to the International society of heart and lung transplant (ISHLT) classifications. For acute cellular rejection, treatment occurs if ≥2R with pulse dose steroids ± thymoglobulin. For antibody-medicated rejection, treatment occurs if ≥ pAMR2 (antibody mediated rejection) and in select cases of pAMR1, usually involving a combination of intravenous immunoglobulin, plasmapheresis, and anti-CD20 therapies. Secondary outcomes included PGD, death, cardiac allograft dysfunction (ejection fraction < 50%), and retransplantation. PGD was defined according to the modified ISHLT criteria.^7^ Moderate PGD was defined as the need for either a postoperative intra-aortic balloon pump or inotrope score >10. Severe PGD was defined as the need for a veno-arterial-extracoporeal membrane oxygenation or ventricular-assist device.

### Statistical analysis

Comparisons were made between DCD and DBD as well as between the different DCD procurement strategies, TA-NRP and DPP. Continuous variables were reported either as mean with standard deviation or as median with interquartile range as appropriate based on normality of distribution. Categorical variables were expressed as counts with percentages. Variables were compared using the unpaired Student's *t*-test and analysis of variance tests. Two-sided *p*-values <0.05 were used. Statistical analyses were completed using R version 4.3.1 2023-06-16 (The R Foundation for Statistical Computing).

## Results

A total of 189 patients underwent HTx between January 2020 and September 2022. Thirteen patients were excluded because of insufficient dd-cfDNA or GEP testing. Of these patients, 9 did not have any testing done, 2 had <6 months of testing, 1 had care transferred to another facility, and 1 passed away in the perioperative period. Twenty patients were excluded because they were multiorgan transplant recipients. Therefore, a total of 156 patients were included in the study with 50 DCD and 106 DBD HTx recipients. Of the DCD HTx recipients, most underwent TA-NRP (37, 74%) compared to DPP (13, 26%) procurement strategies.

Baseline characteristics were similar between DCD and DBD HTx recipients including mean age (58.5 vs 56.9 years, *p* = 0.48), male sex (82% vs 76%, *p* = 0.56), and Caucasian race (46% vs 43%, *p* = 0.78) (Table 1). The predominant etiology of cardiomyopathy was nonischemic (62% vs 59%, *p* = 0.94). There were no significant differences in baseline panel reactive antibody (6% vs 8%, *p* = 0.46) and total ischemic time (224.3 vs 217.2 minutes, *p* = 0.55). There were no significant differences in mean AlloSure at 6 and 12 months between DCD and DBD HTx groups. However, AlloMap was significantly lower at 6 months (*p* = 0.04), but not at 12 months between DCD and DBD HTx groups (Table 1). There were no significant differences in baseline characteristics and mean AlloMap or AlloSure at 6 and 12 months between TA-NRP and DPP DCD HTx groups (Table 2).

**Table 1:** HTx Recipient Characteristics and Outcomes Stratified by DCD and DBD

Recipient characteristics/outcomes		Total (*n* = 156)	DCD (*n* = 50)		DBD (*n* = 106)		*p*-value
Age, mean years (sd)	156	57.4 (13.4)	50	58.5 (12.8)	106	56.9 (13.7)	0.48
Male, no. (%)	156	122 (78)	50	41 (82)	106	81 (76)	0.56
Body mass index, mean mg/k^2^ (sd)	156	27.4 (4.4)	50	28.5 (3.8)	106	26.8 (4.5)	0.03
Race							
White/Caucasian, no. (%)	156	69 (44)	50	23 (46)	106	46 (43)	0.78
Black, no. (%)	156	22 (14)	50	9 (18)	106	13 (12)	
Hispanic, no. (%)	156	40 (26)	50	12 (24)	106	28 (26)	
Asian, no. (%)	156	14 (9)	50	3 (6)	106	11 (10)	
Other, no. (%)	156	11 (7)	50	3 (6)	106	8 (8)	
Blood type							
A, no. (%)	156	49 (31)	50	14 (28)	106	35 (33)	0.11
AB, no. (%)	156	9 (6)	50	2 (4)	106	7 (7)	
B, no. (%)	156	21 (14)	50	3 (6)	106	18 (17)	
O, no. (%)	156	77 (49)	50	31 (62)	106	46 (43)	
PRA, %, (sd)	156	7 (19)	50	6 (15)	106	8 (20)	0.46
Etiology of cardiomyopathy							
Ischemic, no. (%)	156	56 (36)	50	17 (34)	106	39 (37)	0.94
Nonischemic, no. (%)	156	94 (60)	50	31 (62)	106	63 (59)	
Congenital, no. (%)	156	6 (4)	50	2 (4)	106	4 (4)	
Total ischemic time, mean minutes (sd)	156	219.5 (68.3)	50	224.3. (85.1)	106	217.2 (59.0)	0.55
AlloSure and AlloMap							
AlloSure high at 6 months, mean % (sd)	152	0.17 (0.16)	50	0.17 (0.15)	102	0.17 (0.17)	0.98
AlloSure high at 12 months, mean % (sd)	152	0.30 (0.50)	48	0.33 (0.51)	104	0.29 (0.50)	0.97
AlloMap high at 6 months, mean (sd)	143	32 (5)	47	31 (5)	96	33 (4)	0.04
AlloMap high at 12 months, mean (sd)	145	35 (4)	45	34 (3)	100	35 (5)	0.91
Primary graft dysfunction							
Moderate, no. (%)	156	22 (14)	50	9 (18)	106	13 (12)	0.48
Severe, no. (%)	156	6 (4)	50	4 (8)	106	2 (2)	0.16
Acute rejection							
Acute rejection at 1 year, no. (%)	156	26 (17)	50	11 (22)	106	15 (14)	0.32
ACR	156	22 (14)	50	10 (20)	106	12 (11)	0.23
AMR	156	10 (6)	50	2 (4)	106	8 (8)	0.62
Mixed	156	6 (4)	50	1 (2)	106	8 (8)	0.71
Allograft dysfunction (EF < 50%), no. (%)	156	14 (8)	50	4 (8)	106	8 (8)	1
CAV							
ISHLT grade ≥2, no. (%)	146	4 (3)	44	0 (0)	102	4 (4)	0.44
Sirolimus at 1 year, no. (%)	156	96 (62)	50	36 (72)	106	60 (57)	0.1
Death, no. (%)	156	1 (1)	50	1 (2)	106	0 (0)	0.69

Abbreviations: ACR, acute cellular rejection; AMR, antibody-mediated rejection; CAV, cardiac allograft vasculopathy; DBD, donation after brain death; DCD, donation after circulatory death; EF, ejection fraction; HTx, heart transplantation; sd, standard deviation; ISHLT, International society of heart and lung transplant; PRA, panel reactive antibody.

**Table 2:** HTx Recipient Characteristics and Outcomes Stratified by TA-NRP and DPP

Recipient characteristics/outcomes	DCD (*n* = 50)		TA-NRP (*n* = 37)		DPP (*n* = 13)		*p*-value
Age, mean years (sd)	50	58.5 (12.4)	45	58.2 (12.9)	13	59.6 (12.9)	0.73
Male, no. (%)	50	41 (82)	45	29 (78)	13	12 (92)	0.48
Body mass index, mean mg/k^2^ (sd)	50	28.5 (3.8)	45	28.5 (3.8)	13	28.3 (3.8)	0.85
Race							
White/Caucasian, no. (%)	50	23 (46)	45	17 (46)	13	6 (46)	0.82
Black, no. (%)	50	9 (18)	45	7 (19)	13	2 (15)	
Hispanic, no. (%)	50	12 (24)	45	8 (22)	13	4 (31)	
Asian, no. (%)	50	3 (6)	45	3 (8)	13	0 (0)	
Other, no. (%)	50	3 (6)	45	2 (5)	13	1 (8)	
Blood type							
A, no. (%)	50	14 (28)	45	9 (24)	13	5 (38)	0.65
AB, no. (%)	50	2 (4)	45	2 (5)	13	0 (0)	
B, no. (%)	50	3 (6)	45	2 (5)	13	1 (8)	
O, no. (%)	50	31 (62)	45	24 (65)	13	7 (54)	
PRA, %, (sd)	50	6 (15)	45	7 (17)	13	0 (0)	0.12
Etiology of cardiomyopathy							
Ischemic, no. (%)	50	17 (34)	45	12 (32)	13	5 (38)	0.64
Nonischemic, no. (%)	50	31 (62)	45	24 (65)	13	7 (54)	
Congenital, no. (%)	50	2 (4)	45	1 (3)	13	1 (8)	
Total ischemic time, mean minutes (sd)	50	224.3 (85.1)	45	234.6 (61.1)	13	194.9 (130.8)	0.15
AlloSure and AlloMap							
AlloSure high at 6 months, mean % (sd)	50	0.17 (0.15)	37	0.16 (0.14)	13	0.20 (0.17)	0.41
AlloSure high at 12 months, mean % (sd)	48	0.33 (0.51)	35	0.32 (0.56)	13	0.35 (0.33)	0.76
AlloMap high at 6 months, mean (sd)	47	31 (5)	34	31 (6)	13	31 (3)	0.76
AlloMap high at 12 months, mean (sd)	45	34 (3)	33	34 (3)	12	35 (3)	0.63
Primary graft dysfunction							
Moderate, no. (%)	50	9 (18)	45	5 (14)	13	4 (31)	0.33
Severe, no. (%)	50	4 (8)	45	3 (8)	13	1 (8)	1
Acute rejection							
Acute rejection at 1 year, no. (%)	50	11 (22)	45	9 (24)	13	2 (15)	0.78
ACR	50	10 (20)	45	8 (18)	13	2 (15)	0.94
AMR	50	2 (4)	45	2 (4)	13	0 (0)	0.97
Mixed	50	1 (2)	45	1 (2)	13	0 (0)	1
Allograft dysfunction (EF < 50%), no. (%)	50	4 (8)	45	3 (8)	13	1 (8)	1
CAV							
ISHLT grade ≥2, no. (%)	44	0 (0)	31	0 (0)	13	0 (0)	1
Sirolimus at 1 year, no. (%)	50	36 (72)	45	28 (76)	13	8 (62)	0.54
Death, no. (%)	50	1 (2)	45	1 (3)	13	0 (0)	1

Abbreviations: ACR, acute cellular rejection; AMR, antibody-mediated rejection; CAV, cardiac allograft vasculopathy; DCD, donation after circulatory death; DPP, direct procurement protocol with normothermic machine perfusion; HTx, heart transplantation; ISHLT, International society of heart and lung transplant; sd, standard deviation; PRA, panel reactive antibody; TA-NRP, thoraco-abdominal normothermic regional perfusion.

With respect to primary clinical outcome at 1 year, there were no significant differences in AR (22% vs 14%, *p* = 0.32) between DCD and DBD HTx recipients (Table 1). Despite higher use of sirolimus in the DCD group, there were no significant differences in CAV, defined by ISHLT grade ≥2 CAV (0 vs 4%, *p* = 0.44). There were no significant differences in severe PGD (8% vs 2%, *p* = 0.16) in the DCD compared to DBD groups. There were no significant differences in cardiac allograft dysfunction (8% vs 8%, *p* = 1) or mortality (2% vs 0%, *p* = 0.69) in DCD compared to DBD groups (Table 1). In the subgroup analysis, there were no significant differences in primary or secondary outcomes between TA-NRP and DPP DCD patients (Table 2).

## Discussion

To our knowledge, this is the first analysis of dd-cfDNA and GEP specifically in DCD HTx patients. Comparing DCD with DBD HTx group, we observed no significant differences in mean dd-cfDNA at either 6 or 12 months as well as AR, CAV, and mortality at 1 year. However, there was a significant decrease in mean GEP at 6 months, but not at 12 months in the DCD cohort. In a subgroup analysis comparing DCD procurement strategies (DPP vs TA-NRP), there were no significant differences in noninvasive rejection biomarkers or clinical outcomes. These results suggest that ischemic reperfusion and reports of increased risk of PGD with DCD do not lead to short-term, subclinical cardiac graft injury assessed by dd-cfDNA or GEP.

Studies demonstrate that DCD expands the HTx donor pool without any compromise in perioperative or early survival. However, less is known about long-term survival and other post-transplant outcomes, including AR. Using the United Network for Organ Sharing database of 296 DCD HTx recipients, Li et al showed that DCD was associated with more AR episodes before discharge (odds ratio 1.47 *p* = 0.048) and hospitalization for AR (odds ratio 2.03, *p* = 0.026) compared to DBD.^4^ However, there was no significant difference in treated AR within 1 year except with propensity matching. In our study, we similarly found no difference in treated AR at 1 year. Treated AR appears to be more clinically relevant than AR before discharge where there may be false positives due to perioperative ischemic injury or AR-related hospitalization if rejection treatment was deferred.

Various mechanisms inherent to DCD have been postulated for increased risk of post-transplant morbidity. First, the necessity of circulatory death introduces an obligatory ischemic time that can lead to graft injury. This along with functional warm ischemic time, machine perfusion time, and total ischemic time can upregulate the inflammatory cascade and lead to AR. In our study, there was no significant difference in total ischemic time between DCD and DBD and we did not find any differences in treated AR between DCD procurement strategies. PGD requiring mechanical circulatory support has also been observed more frequently in DCD compared to DBD HTx patients.^1^ Use of mechanical circulatory support, especially extracoporeal membrane oxygenation, has been hypothesized to cause an inflammatory response that can activate the innate immune system with downstream consequences of AR or CAV.^4^ We did not observe a significant difference in severe PGD between DCD and DBD HTx cohorts, which may have affected our results.

Noninvasive biomarkers, such as dd-cfDNA and GEP, may provide more mechanistic insights into subclinical graft injury in DCD HTx recipients. Dd-cfDNA is increased immediately after HTx due to allograft ischemia and reperfusion injury and normalizes usually within 1 week.^5^ Therefore, persistent elevations in dd-cfDNA may be harbingers of post-transplant morbidity and studies have shown that an increase in dd-cfDNA can occur months before biopsy-proven AR.^8^ In our study, we did not find any significant differences in dd-cfDNA. There was a significant numerical decrease in GEP at 6 months, but not at 12 months in the DCD cohort. However, this is likely not clinically significant. Our study suggests that there are no significant differences in biomarkers of subclinical cardiac allograft injury at 1 year in the DCD cohort which correlates with post-transplant outcomes. Baseline thresholds for these noninvasive biomarkers appear equivalent in DCD and DBD HTx patients.

### Limitations

The main study limitation is the single-center design and small sample size. Furthermore, there were 13 patients who either had none or insufficient HeartCare testing, which may introduce selection bias. It should also be noted that comparisons between DCD procurement strategies are likely underpowered. These limit the external validity of our study.

## Conclusions

In this single-center study, there were no significant differences in mean dd-cfDNA or treated AR at 1 year between DCD and DBD HTx recipients. There was a significant increase in mean GEP at 6 months, but not 12 months in the DBD cohort. The ischemia reperfusion insult inherent to DCD does not appear to cause significant cardiac allograft injury, assessed using these noninvasive biomarkers. Further large studies are needed to confirm these findings and to evaluate potential differences in DCD procurement strategies.

## Disclosure statement

Victor Pretorius and Eric Adler have served on the advisory board and have received speaker fees for Abbott and Medtronic. None of the other authors has a financial relationship with a commercial entity that has an interest in the subject of the presented manuscript or other conflicts of interest to disclose.

Acknowledgments: None.
